# Augmenting ideational fluency in a creativity task across multiple transcranial direct current stimulation montages

**DOI:** 10.1038/s41598-021-85804-3

**Published:** 2021-04-23

**Authors:** Evangelia G. Chrysikou, Hannah M. Morrow, Austin Flohrschutz, Lauryn Denney

**Affiliations:** 1 Department of Psychology, Drexel University, 3201 Chestnut St., Philadelphia, PA 19140 USA; 2 University of Connecticut, Storrs, USA; 3 University of Arizona, Tucson, USA; 4 University of Kansas Medical Center, Kansas City, USA

**Keywords:** Cognitive control, Problem solving, Cognitive neuroscience

## Abstract

Neuroimaging and transcranial direct current stimulation (tDCS) research has revealed that generating novel ideas is associated with both reductions and increases in prefrontal cortex (PFC) activity, and engagement of posterior occipital cortex, among other regions. However, there is substantial variability in the robustness of these tDCS‐induced effects due to heterogeneous sample sizes, different creativity measures, and methodological diversity in the application of tDCS across laboratories. To address these shortcomings, we used twelve different montages within a standardized tDCS protocol to investigate how altering activity in frontotemporal and occipital cortex impacts creative thinking. Across four experiments, 246 participants generated either the common or an uncommon use for 60 object pictures while undergoing tDCS. Participants also completed a control short-term memory task. We applied active tDCS for 20 min at 1.5 mA through two 5 cm × 5 cm electrodes over left or right ventrolateral prefrontal (areas F7, F8) or occipital (areas O1, O2) cortex, concurrent bilateral stimulation of these regions across polarities, or sham stimulation. Cathodal stimulation of the left, but not right, ventrolateral PFC improved fluency in creative idea generation, but had no effects on originality, as approximated by measures of semantic distance. No effects were obtained for the control tasks. Concurrent bilateral stimulation of the ventrolateral PFC regardless of polarity direction, and excitatory stimulation of occipital cortex did not alter task performance. Highlighting the importance of cross-experimental methodological consistency, these results extend our past findings and contribute to our understanding of the role of left PFC in creative thinking.

## Introduction

Creativity is the ability to generate ideas that are both novel and useful^1^. A recent explosion in cognitive neuroscience research on creativity has elicited important findings toward our understanding of the neural bases of creative thinking from studies employing various neuroscience methodologies, including structural and functional magnetic resonance imaging (fMRI), neuropsychological, and noninvasive brain stimulation experiments^2^. These investigations have generally endorsed a two-stage model of creative thinking according to which creativity entails an initial process of idea generation, followed by a process of selection, elaboration, and refinement of the ideas produced and their evaluation in context. An emerging pattern of results across these studies suggests that cognitive control—the set of regulatory operations supported by prefrontal cortex (PFC)—is critical for each stage of the creative processes through the application of flexible filtering of task-relevant information during creative response generation^3–7^.

A growing literature within creativity neuroscience has employed transcranial direct current stimulation (tDCS), typically over PFC regulatory regions, to augment creative performance. TDCS is a noninvasive brain stimulation methodology that alters cortical excitability through the application of small electric currents (1–2 mA) via electrodes on the scalp^8,9^. Conventionally, relative to placebo or ‘sham’ stimulation, during and immediately following the application of tDCS, anodal stimulation is thought to elicit cortical excitation in the area underling the electrode due to neuron soma membrane depolarization, whereas cathodal stimulation is thought to elicit cortical inhibition in the area underling the electrode due to neuron soma membrane hyperpolarization^8–10^, although these effects might be tDCS-intensity dependent^11^ and substantially more complex neurophysiologically^12–14^.

The use of tDCS for the enhancement of creative cognition has elicited diverse results that point to both the potential but also the substantial variability in the success of tDCS interventions depending on the type of stimulation used (anodal, cathodal, or both), the neural target of tDCS (mainly across bilateral PFC or anterior temporal cortex regions), and the particular requirements of the creativity task employed. Critically, interactions among a creativity task’s reliance on generative or selective processes and tDCS application parameters (location, polarity) can determine the presence of positive effects of tDCS (for recent comprehensive reviews of this literature see^15,16^). As a result, despite the potential of tDCS for the augmentation of creative cognition, comparisons across studies are difficult. Further, an evaluation of this literature suggests that the differences in robustness of tDCS‐induced cognitive enhancements for creativity may be attributed to heterogeneous sample sizes and substantial methodological diversity in the application of tDCS interventions across laboratories, including differences in devices, electrode sizes, strength of stimulation current, stimulation duration, and use of various control tasks^12,17–20^.

To address these issues in past literature, here, we aimed to examine the impact of tDCS on creative ideation using a consistent methodological paradigm across four experiments employing twelve different tDCS montages that were informed directly by our prior fMRI and tDCS work on creative thinking. The selection of tDCS targets was guided by past neuroimaging findings^21^ from a paradigm using a version of the Alternative Uses Task, a standard measure of creative thinking. In the version of the task used in our functional magnetic resonance imaging (fMRI) study, participants in a between-subjects design were tasked with generating either the common or an uncommon use for everyday objects (e.g., using a brick for building vs. using a brick as a hammer), along with a perceptual baseline task, while undergoing fMRI^21^. The results revealed a dissociation between left ventrolateral PFC that was more involved in common use generation relative to baseline, and lateral occipitotemporal cortex that was more involved in uncommon use generation relative to baseline, a finding suggestive of a potential tradeoff between cognitive control and posterior brain systems during creative thinking^6,21–23^. We have further shown that inhibiting the left inferior lateral PFC using tDCS elicited considerable increases in the speed and fluency in which participants generated ideas for uncommon, but not common, uses on this task. In contrast, inhibiting the right PFC or sham stimulation did not influence performance on either task^24^—although other studies have suggested the possible involvement of right frontotemporal regions in creative thinking, including for insight^25–27^ and other problem solving^28,29^ tasks.

Based on these findings, the aim of the present study was to examine the impact of different tDCS interventions to alter activity in the ventrolateral PFC and occipital brain regions shown to be important for creative idea generation in this version of the Alternative Uses task. Our objective was to examine the causal influence of increases or decreases in activity in these regions for creative performance using a comprehensive methodological paradigm across experiments. Contrary to all past tDCS research on creativity, and to allow valid comparisons across our experiments, we maintained equivalent sample sizes across conditions that were recruited from the same participant population, as well as retained the same stimulation device, electrodes, current strength, current duration, and experimental and control tasks across studies. Our selection of montages per experiment was guided by our past work and included: (a) inhibition of the left or right ventrolateral PFC or sham stimulation (L-, R-, sham; Experiment 1; as in^24^); (b) excitation of the left or right ventrolateral PFC or sham stimulation (L + , R + , sham; Experiment 2); (c) inhibition of the left with concurrent excitation of the right ventrolateral PFC, the reverse montage of inhibition of the right with concurrent excitation of the left ventrolateral PFC, or sham stimulation (L-R + , R-L + , sham; Experiment 3); and (d) excitation of the left or right occipital cortex or sham stimulation (L + O1, R + O2, sham Experiment 4; in line with^21^). Electrical field modeling was used as a confirmatory tool and verified the specificity of the stimulation montages over ventrolateral PFC or occipital cortex for all experiments (cf.^24^). Sample size was determined based on the medium-to-large effect size observed in our past tDCS study using this task^24^, but with the aim to improve power by increasing the number of subjects per condition relative to this past experiment by approximately 20%. To provide continuity with our past studies that served as the foundation for our predictions, we used the version of the Alternative Uses task employed in our past work^24^ that presents participants with 60 images of everyday objects and asks them to generate either a single common or a single uncommon use for each. This version of the task requires a single response per item to satisfy experimental neuroscience research constraints, while presenting participants with 4–5 times the number of items included in a typical AUT paradigm for a thorough evaluation of response fluency and originality. Thus, we operationalized creative performance according to two objective metrics of ideational fluency: voice-onset reaction times (RTs) and number of omissions out of the total number of items presented. These metrics provide two unbiased ways to measure the ease of idea generation and for multiple object stimuli, which further increases the task’s ecological validity. We also operationalized originality objectively as captured by semantic distance. Semantic distance is a measure of response originality that is founded on the associative theory of creativity^30^, according to which responses that are farther in semantic space are reflective of a novel, task-appropriate recombination of ideas in semantic memory^31^. Although not a typical measure of response originality, semantic distance captures conceptual remoteness of the ideas generated, thus, serving as an objective proxy for more traditional originality assessments. We chose to focus exclusively on these objective measures of creative performance to avoid problems with subjective creativity assessments that have been noted to impact significantly neuroscience studies on creativity^32,33^. Based on our past findings, we predicted that inhibition of the left but not right ventral PFC or sham stimulation would facilitate creative performance for the unusual use generation task as captured by our objective creativity measures of fluency, but have no impact on the common use generation task, replicating prior work^24^. We further predicted that increases in originality, as captured by semantic distance, would also be observed—although past work using subjective measures has not consistently reported originality effects following tDCS^15^. If inhibition of left relative to right ventrolateral PFC were to benefit creative performance, we anticipated that excitation of the same regions might lead to the opposite effects. In line with past investigations of tDCS for creative thinking using bilateral montages^29,34^, we further hypothesized that the beneficial effect of left ventrolateral PFC inhibition might be amplified with the concurrent excitation of the right ventrolateral PFC for the generation of uncommon (but not common) uses. Lastly, we anticipated that excitation of either the left or the right occipital cortex might benefit performance on this task, given the increased engagement of these regions during creative ideation in our past neuroimaging findings^21^. We used the forward digit span (FDS), a brief working memory assessment, as our negative control task because it engages more dorsal regions of the left PFC than the ones stimulated in the present experiment^35^. Accordingly, if tDCS indeed elicits effects that are regionally specific and does not induce global (e.g., attentional) cognitive changes, it should not lead to any measurable consequences for FDS performance across our montages. Thus, no effects of tDCS were expected for the FDS task.

## Experiment 1

The aim of Experiment 1 was to replicate and extend our past work on the effects of inhibiting the left or right ventrolateral PFC relative to sham stimulation on the fluency and originality of participants’ responses on the AUT^24^. We anticipated that inhibition of the left, but not, right ventrolateral PFC would elicit benefits for ideational fluency, which might also extend to benefits for originality as measured by semantic distance.

## Methods

### Participants

Sixty (*N* = 60) right-handed, native English speakers (mean age = 19.20; 24 males) participated in the study for course credit after providing informed consent. Across all experiments, participants were excluded if they met criteria for contraindications for tDCS (e.g., pregnancy [as confirmed by urine test], history of seizures or head trauma). The studies were approved by the University of Kansas Institutional Review Board. All methods were carried out in accordance with relevant guidelines and regulations.

### Materials

We used a modified computerized version of the Alternative Uses Task from Chrysikou et al.^24^, in which participants are shown pictures of common items (e.g., belt) and they are asked to generate verbally either the common (CU) or an uncommon use (UU) for each. Sixty greyscale pictures (448 × 336 ppi) of everyday objects were randomly presented on a gray background for 9000 ms each with 3000 ms interstimulus interval via E-Prime software on a Mac computer running Windows. Voice-onset RTs were recorded with a microphone integrated with E-Prime. Participant responses were also recorded via Audacity software on a Mac laptop computer for later assessment of task compliance and response originality. The FDS, a brief working memory assessment, was used as a negative control task. In the FDS, participants are presented with 16 strings of numbers of increasing length and they are asked to repeat each of these strings back to the experimenter, who is positioned outside of the participants’ field of view. Performance on the task is measured as total number of correct trials on the task.

### Design and procedure

Participants were randomly assigned to perform either the UU or the CU task under one of three stimulation conditions: (1) cathodal tDCS over left ventrolateral PFC (area F7 in the 10/20 electroencephalogram [EEG] system); (2) cathodal stimulation over right ventrolateral PFC (area F8 in the 10/20 EEG system), or (3) sham stimulation. Task order was counterbalanced with the FDS task. Participants in the CU task were asked to generate the common, everyday use for each object (e.g., a belt is used to hold one’s pants up), whereas participants in the UU task were asked to generate a novel use for each object (e.g., using a belt as a tourniquet). Participants were instructed to remain silent if unable to generate a response for a particular object. For the FDS control task participants were asked to repeat each string of numbers in the order that were read to them. Before the experiment began, participants underwent a brief training to become familiar with the tasks.

### tDCS parameters

TDCS was administered in a single-blind design using a NeuroConn DC-Stimulator Plus (NeuroConn, GmbH, http://www.neuroconn.de/dc-stimulator_en/) at 1.5 mA via two 5 cm × 5 cm electrodes (current density = 0.06 mA/cm^2^) for a maximum of 20 min, including 10 s ramp-up and 10 s ramp-down time. The electrodes were placed into saline-soaked sponges (4 mL of saline per sponge side applied through a syringe) and held in place with rubber straps. The stimulation target was guided by prior work^21,24^ to be in the ventrolateral PFC and was determined as either area F7 (L-, *n* = 20) or F8 (R-, *n* = 20)^36^ depending on the participant’s condition; the anode (i.e., reference electrode) was placed over the contralateral mastoid. The stimulation site was identified with a BraiNet 10/20 Placement cap (https://bio-medical.com/) and was marked on the participant’s scalp with a marker. In the active conditions, stimulation began for 90 s prior to the experimental or control task while participants viewed a blank screen to allow for tDCS to create the hyperpolarizing changes to the underlying cortex prior to task onset. Participants in the sham condition (sham, *n* = 20) were stimulated over F7 or F8 (counterbalanced across subjects) under the above parameters for 90 s with the same ramp rates to create the sensation of receiving tDCS as in the experimental conditions upon which stimulation was interrupted unbeknownst to the subjects; 90 s of stimulation was considered short enough time such that there would be no significant lasting changes in excitability of the underlying neuronal population that would elicit reliable task effects.

## Results

### Overview & Scoring

Across all 4 experiments, participant responses were transcribed and manually inspected for compliance with task instructions per condition, with less than 1% of erroneous responses removed. There were no task order effects, hence, all results are reported collapsed across task order for all experiments. For the UU and CU tasks, median voice-onset reaction times for each participant were collected and averaged across subjects in each condition for all experiments. Similarly, the number of omissions for the UU and CU tasks was collected for each participant and averaged across subjects in each condition for all experiments. Response originality was scored according to semantic distance, a computational method that employs natural language processing to quantify the semantic relatedness of texts. Specifically, we followed the SemDis approach that relies on a latent semantic distance factor—comprised of the common variance from five semantic models—and which has been shown to correlate with subjective judgments of novelty in creativity tasks^37^. We used the automated SemDis platform (semdis.wlu.psu.edu) to compute the semantic distance between each item and each participant response across all participants and conditions, separately for each experiment. We used all five spaces incorporated in SemDis for the semantic distance calculations; according to this method, the cosine angle between the word vectors represents semantic similarity; semantic distance is then computed by subtracting this similarity from 1^31,37^. Latent variable modeling was then used to extract the common variance from the five semantic models, which has been shown to benefit the reliability and generalizability of the results relative to single model approaches^37^. We then calculated an average semantic distance score across all valid responses for the experimental stimuli for each participant in each experimental condition, which was then used for the statistical analysis. Finally, for the FDS task the number of correct responses (out of a total of 16) was collected for each participant and averaged across subjects for all studies.

### Voice-onset reaction times

**Figure 1:**
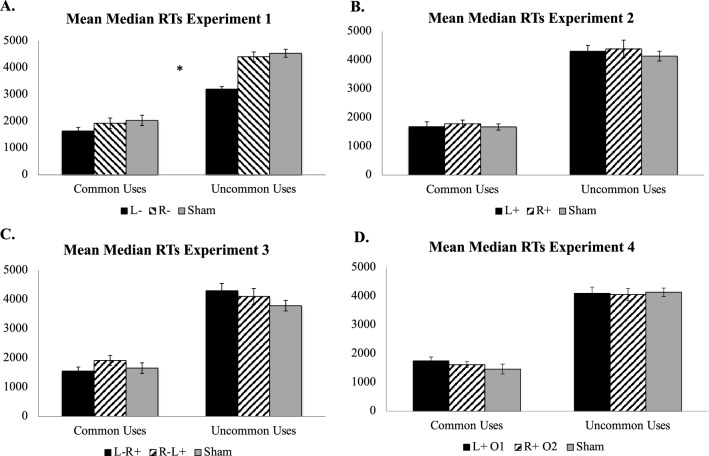
Voice onset mean median RT results for Experiments 1–4 by task and stimulation condition. (**A**) Voice onset mean median RTs for Experiment 1; (**B**) Voice onset mean median RTs for Experiment 2; (**C**) Voice onset mean median RTs for Experiment 3; (**D**) Voice onset mean median RTs for Experiment 4; L- = cathodal tDCS over left ventrolateral PFC; R- = cathodal tDCS over right ventrolateral PFC. L +  = anodal tDCS over left ventrolateral PFC; R +  = anodal tDCS over right ventrolateral PFC. L-R +  = cathodal tDCS over left ventrolateral PFC with concurrent anodal tDCS over right ventrolateral PFC; R-L +  = cathodal tDCS over right ventrolateral PFC with concurrent anodal tDCS over left ventrolateral PFC. L + O1 = anodal tDCS over left occipitotemporal cortex; R + O2 = anodal tDCS over right occipitotemporal cortex. RT = Reaction Times. The asterisk [*] represents *p* < .01 for the significant task × stimulation condition interaction for voice-onset RTs for Experiment 1.

For Experiment 1, there was a significant main effect of task (*F*[1, 2] = 49.61, *p* = 0.02, *η*^2^ = 0.96) but no main effect of stimulation condition (*F*[2, 2] = 3.04, *p* = 0.25, *η*^2^ = 0.75; Fig. 1A). Importantly, there was a significant task × stimulation condition interaction (*F*[2,54] = 5.58, *p* = 0.006; *η*^2^ = 0.17). Post hoc Tukey’s Honestly Significant Difference (HSD) tests showed that for the UU task participants who received cathodal stimulation over left ventrolateral PFC (L-) generated responses significantly faster relative to participants who received cathodal stimulation over right ventrolateral PFC (R-, *p* < 0.001) or sham stimulation (*p* < 0.001), who did not differ from each other (*p* = 0.99). Post hoc comparisons for the CU task were not significant (all *p*s > 0.55).

### Response omissions

**Figure 2:**
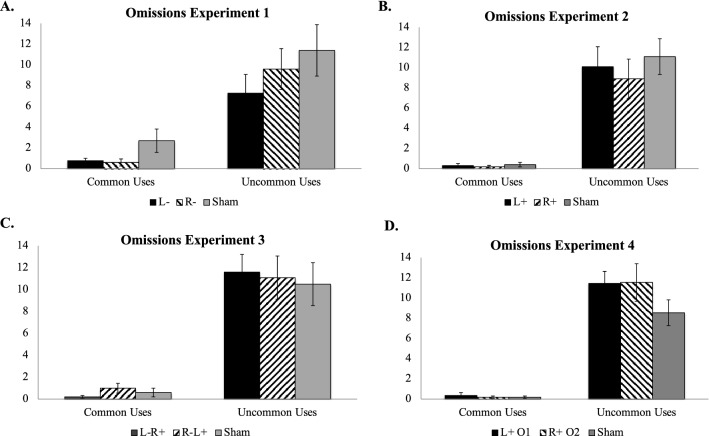
Average number of response omissions for Experiments 1–4 by task and stimulation condition. (**A**) Average number of response omissions for Experiment 1; (**B**) Average number of response omissions for Experiment 2; (**C**) Average number of response omissions for Experiment 3; (**D**) Average number of response omissions for Experiment 4; L- = cathodal tDCS over left ventrolateral PFC; R- = cathodal tDCS over right ventrolateral PFC. L +  = anodal tDCS over left ventrolateral PFC; R +  = anodal tDCS over right ventrolateral PFC. L-R +  = cathodal tDCS over left ventrolateral PFC with concurrent anodal tDCS over right ventrolateral PFC; R-L +  = cathodal tDCS over right ventrolateral PFC with concurrent anodal tDCS over left ventrolateral PFC. L + O1 = anodal tDCS over left occipitotemporal cortex; R + O2 = anodal tDCS over right occipitotemporal cortex.

There was a significant difference in number of omitted responses between UU and CU tasks (*F*[1, 2] = 104.62, *p* = 0.009; *η*^2^ = 0.98; Fig. 2A), but there was no main effect of stimulation condition (*F*[2, 2] = 5.04, *p* = 0.17; *η*^2^ = 0.83) or a significant task × stimulation condition interaction (*F*[2,54] = 0.37, *p* = 0.69; *η*^2^ = 0.01). Post hoc Tukey’s HSD tests showed that for the UU task participants who received cathodal stimulation over left ventrolateral PFC (L-) generated significantly more responses relative to participants who received sham stimulation (*p* = 0.04) but not relative to those who received cathodal stimulation over right ventrolateral PFC (R-, *p* = 0.99), who did not differ from each other (*p* = 0.06). Post hoc comparisons for the CU task were not significant (all *p*s > 0.95).

### Response originality

**Figure 3:**
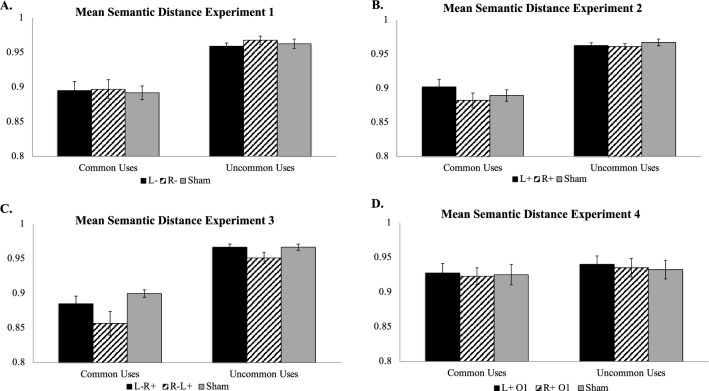
Mean semantic distance for Experiments 1–4 by task and stimulation condition. (**A**) Mean semantic distance for Experiment 1; (**B**) Mean semantic distance for Experiment 2; (**C**) Mean semantic distance for Experiment 3; (**D**) Mean semantic distance for Experiment 4; L- = cathodal tDCS over left ventrolateral PFC; R- = cathodal tDCS over right ventrolateral PFC. L +  = anodal tDCS over left ventrolateral PFC; R +  = anodal tDCS over right ventrolateral PFC. L-R +  = cathodal tDCS over left ventrolateral PFC with concurrent anodal tDCS over right ventrolateral PFC; R-L +  = cathodal tDCS over right ventrolateral PFC with concurrent anodal tDCS over left ventrolateral PFC. L + O1 = anodal tDCS over left occipitotemporal cortex; R + O2 = anodal tDCS over right occipitotemporal cortex.

Verbal response data for the UU task from three subjects (one per stimulation condition) were lost due to a recording error, leaving 57 participants for analysis. There was a significant difference in the semantic distance of the generated responses between UU and CU tasks (*F*[1, 2] = 963.37, *p* = 0.001; *η*^2^ = 1.00; Fig. 3A), which confirms participants in the UU condition were generating responses consistent with the UU task. In contrast, there was no main effect of stimulation condition (*F*[2, 2] = 2.43, *p* = 0.29; *η*^2^ = 0.71) or a significant task × stimulation condition interaction (*F*[2,51] = 0.77, *p* = 0.93; *η*^2^ = 0.003). Post hoc Tukey’s HSD tests comparisons for either the UU or the CU task were not significant (all *p*s > 0.85).

### Forward digit span

**Figure 4:**
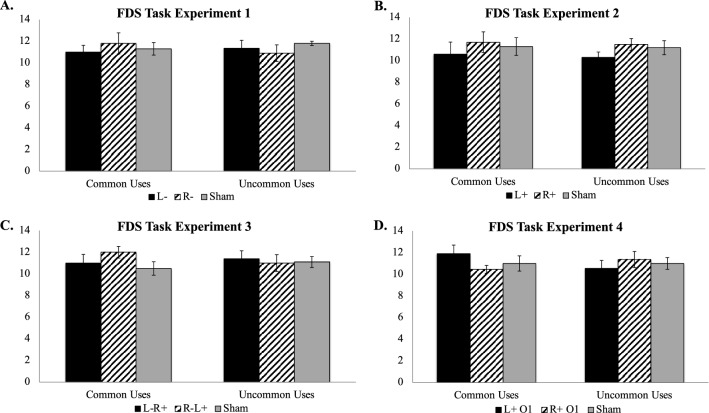
Forward Digit Span (FDS) task mean accuracy for Experiments 1–4 by task and stimulation condition. (**A**) FDS task mean accuracy for Experiment 1; (**B**) FDS task mean accuracy for Experiment 2; (**C**) FDS task mean accuracy for Experiment 3; (**D**) FDS task mean accuracy for Experiment 4; L- = cathodal tDCS over left ventrolateral PFC; R- = cathodal tDCS over right ventrolateral PFC. L +  = anodal tDCS over left ventrolateral PFC; R +  = anodal tDCS over right ventrolateral PFC. L-R +  = cathodal tDCS over left ventrolateral PFC with concurrent anodal tDCS over right ventrolateral PFC; R-L +  = cathodal tDCS over right ventrolateral PFC with concurrent anodal tDCS over left ventrolateral PFC. L + O1 = anodal tDCS over left occipitotemporal cortex; R + O2 = anodal tDCS over right occipitotemporal cortex.

As predicted, there were no significant differences in FDS performance among the stimulation conditions (*F*[2, 2]) = 0.23, *p* = 0.82; *η*^2^ = 0.19; Fig. 4A). Post hoc Tukey’s HSD comparisons further did not reveal any significant pairwise differences between stimulation conditions (all *p*s > 0.97).

## Experiment 2

Experiment 2 aimed to complement the findings of Experiment 1, by examining the effects of excitation of the left or right ventrolateral PFC relative to sham stimulation for fluency and originality as captured by semantic distance. Following the findings of Experiment 1, we hypothesized that excitation of the left ventrolateral PFC would lead to possible deficits for the UU but not the CU task, whereas excitation of the right ventrolateral PFC might elicit the opposite effects.

## Methods

### Participants

Sixty (*N* = 60) right-handed, native English speakers (mean age = 18.83; 21 males) participated in the study for course credit after providing informed consent. None of the subjects had taken part in Experiment 1.

### Materials

The materials were identical to Experiment 1.

### tDCS parameters, design, and procedure

The design and procedures were identical to Experiment 1 with the exception that the participants were randomly assigned to perform either the CU or the UU tasks under the following stimulation conditions: (1) excitation of the left ventrolateral PFC (anode over F7 with cathode over contralateral mastoid; L + , *n* = 20); (2) excitation of the right ventrolateral PFC (anode over F8 with cathode over contralateral mastoid; R + , *n* = 20); or (3) sham stimulation using the active conditions electrode configuration for Experiment 2, counterbalanced across subjects (sham, *n* = 20).

## Results

All coding and data analyses procedures were identical to Experiment 1.

### Voice-onset reaction times

For Experiment 2, there was a significant main effect of task (*F*[1, 2] = 270.08, *p* < 0.001, *η*^2^ = 0.83; Fig. 1B), but no main effect of stimulation condition (*F*[2, 2] = 046, *p* = 0.63, *η*^2^ = 0.02) and no task × stimulation condition interaction (*F*[2,54] = 0.10, *p* = 0.90; *η*^2^ = 0.004). Post hoc Tukey’s HSD comparisons did not reveal any significant pairwise comparisons neither for the UU nor for the CU task (all *p*s > 0.60).

### Response omissions

There was a significant difference in number of omitted responses between UU and CU tasks (*F*[1, 2] = 77.92, *p* < 0.001; *η*^2^ = 0.59; Fig. 2B), but there was no main effect of stimulation condition (*F*[2, 2] = 0.40, *p* = 0.68; *η*^2^ = 0.01) or a significant task × stimulation condition interaction (*F*[2,54] = 0.28, *p* = 0.76; *η*^2^ = 0.01). Post hoc Tukey’s HSD comparisons did not reveal any significant pairwise comparisons neither for the UU nor for the CU task (all *p*s > 0.65).

### Response originality

There was a significant difference in the semantic distance of the generated responses between UU and CU tasks (*F*[1, 2] = 126.38, *p* < 0.001; *η*^2^ = 0.71; Fig. 3B), which confirms participants in the UU condition were generating responses consistent with the UU task. In contrast, there was no main effect of stimulation condition (*F*[2, 2] = 0.91, *p* = 0.41; *η*^2^ = 0.03) or a significant task × stimulation condition interaction (*F*[2,54] = 0.83, *p* = 0.44; *η*^2^ = 0.03). Post hoc Tukey’s HSD tests comparisons for either the UU or the CU task were not significant (all *p*s > 0.69).

### Forward digit span

As predicted, there were no differences in FDS performance among the stimulation conditions (*F*[2, 2]) = 1.16, *p* = 0.32; *η*^2^ = 0.04; Fig. 4B). Post hoc Tukey’s HSD comparisons further did not reveal any significant pairwise differences between stimulation conditions (all *p*s > 0.31).

## Experiment 3

The aim of Experiment 3 was to extend the findings of the prior two studies by testing whether inhibition of the left with concurrent excitation of the right ventrolateral PFC would benefit AUT performance relative to the reverse montage of inhibition of the right with concurrent excitation of the left ventrolateral PFC, or sham stimulation. Consistent with past research that has explored the impact of bilateral montages for creative thinking^29,34^, we predicted that the beneficial effect of left ventrolateral PFC inhibition might be amplified with the concurrent excitation of the right ventrolateral PFC for the generation of uncommon (but not common) uses.

## Methods

### Participants

Sixty (*N* = 60) right-handed, native English speakers (mean age = 19.02; 25 males) participated in the study for course credit after providing informed consent. None of the subjects had taken part in Experiments 1 or 2.

### Materials

The materials were identical to Experiment 1.

### tDCS parameters, design, and procedure

The design and procedures were identical to Experiment 1 with the exception that the participants were randomly assigned to perform either the CU or the UU tasks under the following stimulation conditions: (1) inhibition of the left ventrolateral PFC with concurrent excitation of the right ventrolateral PFC (cathode over F7 with anode under F8; L-R + , *n* = 20); (2) inhibition of the right ventrolateral PFC with concurrent excitation of the left ventrolateral PFC (cathode over F8 with anode under F7; R-L + , *n* = 20); or (3) sham stimulation using the active conditions electrode configuration for Experiment 3, counterbalanced across subjects (sham, *n* = 20).

## Results

All coding and data analyses procedures were identical to Experiment 1.

### Voice-onset reaction times

For Experiment 3, there was a significant main effect of task (*F*[1, 2] = 148.40, *p* = 0.007, *η*^2^ = 0.99; Fig. 1C), no main effect of stimulation condition (*F*[2, 2] = 0.81, *p* = 0.55, *η*^2^ = 0.45), and no task × stimulation condition interaction (*F*[2,54] = 1.35, *p* = 0.27; *η*^2^ = 0.05). Post hoc Tukey’s HSD comparisons did not reveal any significant pairwise comparisons neither for the UU nor for the CU task (all *p*s > 0.30).

### Response omissions

There was a significant main effect of task (*F*[1, 2] = 495.46, *p* = 0.002; *η*^2^ = 1.00), but there was no main effect of stimulation condition (*F*[2, 2] = 0.40, *p* = 0.72; *η*^2^ = 0.28) or a significant task × stimulation condition interaction (*F*[2,54] = 0.19, *p* = 0.83; *η*^2^ = 0.007; Fig. 2C). Post hoc Tukey’s HSD comparisons did not reveal any significant pairwise comparisons neither for the UU nor for the CU task (all *p*s > 0.27).

### Response originality

Verbal response data for the UU task from one subject in the L + R- condition were lost due to a recording error, leaving 59 participants for analysis. There was a significant difference in the semantic distance of the generated responses between UU and CU tasks (*F*[1, 2] = 106.52, *p* = 0.009; *η*^2^ = 0.98; Fig. 3C), which confirms participants in the UU condition were generating responses consistent with the UU task. In contrast, there was no main effect of stimulation condition (*F*[2, 2] = 4.94, *p* = 0.17; *η*^2^ = 0.83) or a significant task × stimulation condition interaction (*F*[2,53] = 0.99, *p* = 0.38; *η*^2^ = 0.04). Post hoc Tukey’s HSD tests comparisons for either the UU or the CU task were not significant (all *p*s > 0.14).

### Forward digit span

As hypothesized, there were no differences in FDS performance among the stimulation conditions (*F*[2, 2]) = 0.65, *p* = 0.61; *η*^2^ = 0.39; Fig. 4C). Post hoc Tukey’s HSD comparisons further did not reveal any significant pairwise differences between stimulation conditions (all *p*s > 0.26).

## Experiment 4

Our past neuroimaging work^21^ has revealed increased occipital cortex activity during the generation of uncommon (but not common) uses during the AUT. Guided by the results of that study, Experiment 4 aimed to examine whether excitation of the left or right occipital cortex with tDCS would lead to benefits for fluency and originality relative to sham stimulation. We anticipated that excitation of either left or right occipital cortex might benefit performance on this task, given the increased engagement of these regions during creative ideation in our past neuroimaging findings^21^.

## Methods

Sixty-six (*N* = 66) right-handed, native English speakers (mean age = 19.68; 29 males) participated in the study for course credit after providing informed consent. None of the subjects had taken part in Experiments 1–3.

### Materials

The materials were identical to Experiment 1.

### tDCS parameters, design, and procedure

The design and procedures were identical to Experiment 1 with the exception that the participants were randomly assigned to perform either the CU or the UU tasks under the following stimulation conditions: (1) excitation of the left lateral occipital cortex (anode over O1 with cathode over contralateral mastoid; L + O1, *n* = 22); (2) excitation of the right lateral occipital cortex (anode over O2 with cathode over contralateral mastoid; R + O2, *n* = 22); or (3) sham stimulation using the active conditions electrode configuration for Experiment 4, counterbalanced across subjects (sham, *n* = 22).

## Results

All coding and data analyses procedures were identical to Experiment 1.

### Voice-onset reaction times

For Experiment 4, there was a significant main effect of task (*F*[1, 2] = 692.95, *p* = 0.001, *η*^2^ = 1.00; Fig. 1D), no main effect of stimulation condition (*F*[2, 2] = 0.63, *p* = 0.62, *η*^2^ = 0.39), and no task × stimulation condition interaction (*F*[2,60] = 0.50, *p* = 0.63; *η*^2^ = 0.02). Post hoc Tukey’s HSD comparisons did not reveal any significant pairwise comparisons neither for the UU nor for the CU task (all *p*s > 0.34).

### Response omissions

Similar to RTs, there was a significant main effect of task (*F*[1, 2] = 115.04, *p* = 0.009; *η*^2^ = 0.98; Fig. 2D), but there was no main effect of stimulation condition (*F*[2, 2] = 1.12, *p* = 0.47; *η*^2^ = 0.53) or a significant task × stimulation condition interaction (*F*[2,60] = 1.25, *p* = 0.30; *η*^2^ = 0.04). Post hoc Tukey’s HSD comparisons did not reveal any significant pairwise comparisons neither for the UU (all *p*s > 0.33) nor for the CU task (all *p*s > 0.77).

### Response originality

There was a significant difference in the semantic distance of the generated responses between UU and CU tasks (*F*[1, 2] = 37.62, *p* = 0.026; *η*^2^ = 0.95; Fig. 3D), which confirms participants in the UU condition were generating responses consistent with the UU task. In contrast, there was no main effect of stimulation condition (*F*[2, 2] = 3.60, *p* = 0.22; *η*^2^ = 0.78) or a significant task × stimulation condition interaction (*F*[2,54] = 0.03, *p* = 0.97; *η*^2^ = 0.001). Post hoc Tukey’s HSD tests comparisons for either the UU or the CU task were not significant (all *p*s > 0.70).

### Forward digit span

There were no significant differences in FDS performance among the stimulation conditions (*F*[2, 2]) = 0.08, *p* = 0.92; *η*^2^ = 0.08; Fig. 4D). Post hoc Tukey’s HSD comparisons did not reveal any significant pairwise differences between stimulation conditions (all *p*s > 0.26).

## Discussion and conclusions

Studies using tDCS for the enhancement of creative performance have reported positive effects of both anodal and cathodal stimulation over PFC regions for creativity. On the other hand, the significant variability in the results across studies—partially attributed to substantial methodological diversity in the application of tDCS across investigations—has made it difficult to reach concrete conclusions regarding the effectiveness of tDCS for the augmentation of creative cognition. To address these concerns, here, we examined the impact of multiple tDCS montages across 4 experiments on a multiple-item, single-response version of the Alternative Uses Task—a classic measure of creative thinking—adapted for experimental neuroscience research. Montage selection was guided by past fMRI work using the same task^21^ and focused on areas of maximal fMRI activation in the left ventrolateral PFC and the lateral occipitotemporal cortex. Our approach differs from all past tDCS research on creativity in that we maintained equivalent sample sizes across conditions that were recruited from the same participant population, and retained the same stimulation device, electrode sizes, current strength and duration, and experimental and control tasks across all 4 studies. To our knowledge, this is the first rigorous multi-study investigation of the effects of tDCS on creative thinking across a large sample of subjects with this level of methodological consistency that can allow for valid comparisons across experiments.

Our results showed that only unilateral cathodal inhibition of the left ventrolateral PFC can improve ideational fluency as measured by participants’ voice-onset RTs, whereas similar—but weaker—results were observed for the number of responses produced. No effects of stimulation were reported for originality, as captured by mean semantic distance. These results replicate our past findings with the same task and stimulation parameters in a smaller sample^24^, as well as other neuroimaging and neurostimulation findings that have reported benefits of left hemisphere hypofrontality for the generative aspects of creativity^5,34,38–41^. Although past work^29,34^ has suggested benefits of increased activity in right PFC for creative ideation, neither unilateral excitation of the right PFC, nor excitation of the right with inhibition of the left PFC elicited significant effects for performance in our studies. We attribute the deviation of our results from this past literature to the differences in the location and size of the tDCS electrodes, as well as differences in the particular creativity measures used^15^. Lastly, despite the robust engagement of lateral occipitotemporal cortex in the uncommon uses task in our fMRI study^21^, enhancing activity in these regions with anodal tDCS did not elicit any measurable benefits to ideational fluency or originality.

The dissociation between the facilitatory effects of left ventrolateral PFC inhibition for ideational fluency and the lack of effects for originality in the version of the AUT employed in this study replicates prior tDCS research that has shown measurable increases in fluency^24,34^ and flexibility^34^, but not originality^24,34^. Although fluency has been argued to be a more objective measure of creativity than originality^42^, the semantic distance approach we employed in this set of experiments to index originality^37^ circumvents the vulnerability of this construct to subjectivity bias. On the other hand, originality can be confounded by fluency^43^. Indeed, the high correlation among fluency, originality, and flexibility further speaks to the view that all three constructs measure similar but distinct aspects of creative thinking^44,45^. It is, thus, likely that different brain regions might contribute differently to each of these aspects of creative thought, with fluency associated with ventrolateral PFC involvement, whereas originality associated with ventral temporal cortex^34,41^. Additional research exploring both neuroanatomically-specific effects and neural oscillations is required for a comprehensive assessment of this proposition.

Alternatively, the benefits of left ventrolateral PFC inhibition for the fluency of creative ideas, but not semantic distance we observed in Experiment 1 are consistent with accounts that suggest a reduction in the evaluative mechanisms supported by this brain region may result in lower inhibition for the ideas produced during the generative phases of the creativity process^24,34,41^. A reduction in the selection mechanisms that determine the salience of generated responses would restrain the dominance of certain ideas relative to others, thus, increasing fluency in the ideas reported. However, such increases in fluency are not necessarily paired with originality gains: A reduction in the evaluative processes involved in creative thinking may render more ideas as possible candidates for generation, but need not increase the semantic distance of the generated responses—an account consistent with prior work on the role of hypofrontality for creative thinking^5–7,41,46,47^.

We note that although our sample size was guided by prior effect sizes and it is increased relative to past studies, the relatively small number of participants by condition is a limitation— though one contextualized by the large number of participants, overall. To allow for continuity with our prior work and to examine the specific involvement of the ventrolateral PFC in creativity idea generation, we employed a multiple-item, single-response measure of the Alternative Uses Task. Because of this choice, we were only able to evaluate response fluency and originality, but not flexibility—a typical aspect of AUT scoring that is predicated on multiple responses per item. Although we are unable to evaluate flexibility in the present set of experiments, additional experimental work is required to determine the impact of changes in ventrolateral PFC excitability for flexibility in creativity tasks^34^. Further, the use of a between-subjects design for the present experiments limits our ability to capture with precision the effects of different montages for each participant. Although within-subjects, multi-session investigations are challenging, they can provide a powerful way to solidify the effects we report here. Lastly, despite the use of current flow models confirming the specificity of our montages, targeting the brain with high-definition tDCS holds significant potential to elicit more robust results in future experiments. Notwithstanding these limitations, our study is the first methodologically rigorous examination of the effectiveness of multiple tDCS montages for creative cognition that supports the potential benefits of transient reductions in ventrolateral PFC activity for creative thinking.
